# A new method for the detection of *Mycobacterium tuberculosis* based on the CRISPR/Cas system

**DOI:** 10.1186/s12879-023-08656-4

**Published:** 2023-10-11

**Authors:** Xiaoyu Zhang, Xiaoying He, Yubo Zhang, Lei Chen, Zhaobao Pan, Yueying Huang, Heng Li

**Affiliations:** 1 Department of Medical Laboratory, Weifang Medical University, Weifang, 261041 Shandong China; 2 Department of Laboratory Medicine, the Second People’s Hospital of Weifang 261041, Weifang, 261041 Shandong China; 3 Department of Laboratory Medicine, Peking University Shenzhen Hospital, Shenzhen, China

**Keywords:** CRISPR, RAA, *Mycobacterium tuberculosis*, Molecular testing, Diagnosis

## Abstract

**Object:**

*Mycobacterium tuberculosis* (MTB) is a bacterium that can cause zoonoses by aerosol transmission. Tuberculosis (TB) caused by MTB heavily burdens world public health security. Developing efficient, specific, convenient, and inexpensive MTB assays are essential for preventing and controlling TB.

**Methods:**

In this study, we established a specific detection method for MTB using the Clustered Regularly Interspersed Short Palindromic Repeats (CRISPR) system, combined with recombinase mediated isothermal nucleic acid amplification (RAA) to improve the sensitivity of the detection system and achieve “two-level” amplification of the detection signal. The sensitivity and specificity of RAA combined with the CRISPR/Cas system were analyzed. Using BACTEC 960 culture as the gold standard for detecting MTB, we established the TB-CRISPR technique by testing 504 samples from patients with suspected TB.

**Results:**

MTB H37Ra could be seen as low as 3.13 CFU/mL by the CRISPR-Cas12a system targeting IS6110. With BACTEC960 culture (120 positives and 384 negatives) as the gold standard, the sensitivity of the TB-CRISPR technique was 0.883 (0.809–0.932), and the specificity was 0.940 (0.910–0.961). According to the receiver operating characteristic (ROC) curve analysis, the area under the curve (AUC) reached 0.944 (0.914–0.975) within 95% CI. The positive likelihood ratio (PLR) was 14.747 (9.870-22.035), and the negative likelihood ratio (NLR) was 0.124 (0.076–0.203). The positive predictive value (PPV) was 0.822 (0.742–0.881), and the negative predictive value (NPV) was 0.963 (0.937–0.979).

**Conclusion:**

TB-CRISPR plays an essential role in the molecular diagnosis of TB. The whole detection time is less than 1.5 h. It is easy to operate and does not need complex instruments. It is of great significance for the rapid detection of MTB and the clinical diagnosis of TB.

**Supplementary Information:**

The online version contains supplementary material available at 10.1186/s12879-023-08656-4.

## Introduction

Tuberculosis (TB) is a chronic infectious disease caused by *Mycobacterium tuberculosis* (MTB) [1]. TB has ranked among the top ten causes of death worldwide since 2007 and is the number one killer among infectious diseases [2]. According to the Global Tuberculosis Report 2021 [3], about 2 billion people worldwide are infected with MTB, and 9.87 million people suffer from TB, with India, China, and Russia having the heaviest burden. The coronavirus disease 2019 (COVID-19) pandemic has significantly impacted TB prevention and control efforts, causing an estimated increase of approximately 100,000 TB deaths worldwide in 2020 due to the interruption of essential TB services. Through the research of mycobacterium tuberculosis detection technology and the development of new anti-tuberculosis drugs, has become the key to the prevention and treatment of tuberculosis.

Etiological detection is of great significance for effectively preventing the TB epidemic and designing clinical chemotherapy regimens. Traditional diagnostic methods for tuberculosis patients include bacteriology, immunology, and molecular biology [4]. Bacteriological clinical diagnosis is the gold standard for diagnosing infectious tuberculosis patients, of which the primary diagnostic methods are acid-fast staining of sputum smear and MTB culture. The traditional TB diagnosis detection methods are mainly smear microscopy and sputum culture. Smear microscopy has low sensitivity and specificity. Although sputum culture is the “gold standard” for diagnosing tuberculosis, it cannot meet the needs of rapid clinical diagnosis due to its long time [5–7]. The combined use of multiple etiological detection methods can significantly improve the efficiency of tuberculosis diagnosis, but the positive detection rate still needs to be determined to improve further. Early, rapid and accurate detection of MTB and its drug resistance has become an urgent problem to be solved in TB prevention and control.

Clustered Regularly Interspersed Short Palindromic Repeats (CRISPR) are adaptive immune systems in bacteria and archaea [8]. CRISPR sequences consist of numerous short and conserved repeat regions and spacers. When foreign genes invade bacteria, the immune defense enters the adaptation stage, and the bacteria obtain foreign genes to insert into their genome. When the foreign gene re-invades, CRISPR sequences transcribe CRISPR RNA (crRNA) and trans-acting crRNA (tracrRNA) under the regulation of the pilot region. tracrRNA complements crRNA to form guide RNA (gRNA), a complex composed of gRNA and Cas proteins to specifically recognize and cut foreign genes for defense [9]. By designing the sequence of gRNA, the gRNA/Cas protein complex can specifically identify foreign fragments, activate the nucleic acid cleaving enzyme activity of Cas protein, and play a role in cleaving unfamiliar pieces in the system [10]. Trans-cleavage movement can amplify the detection signal after adding reporter probes modified by the fluorescence and quenching groups to the reaction system to achieve real-time detection of specific targets [11–13].

With the rapid development of molecular biology detection technology, CRISPR/Cas system for pathogen detection has been widely used in clinical practice. CRISPR/Cas system shows highly high specificity and sensitivity [14]. In this study, we established an MTB identification method TB-CRISPR based on CRISPR/Cas system combined with recombinase aided amplification (RAA). RAA is an emerging isothermal nucleic acid amplification technology that utilizes three enzymes: recombinase (UvsX), single chain binding protein (SSB), and DNA polymerase (Klenow). At the optimal temperature of 37 °C, DNA fragments can be amplified within 5–20 min, thus achieving efficient DNA amplification [15]. By comparing the BACTEC960 culture results with 504 clinical samples, we evaluated the detection effect of TB-CRISPR in tuberculosis and further analyzed its application value in tuberculosis prevention and control.

## Materials and methods

### Expression and purification of Cas12a protein

The pMBP-LbCas12a recombinant plasmid used in this study was purchased from Addgene Global Plasmid Information Sharing Platform (Plasmid#113,431) and was 9653 bp in size. The recombinant plasmid was transformed into DH5α competent state and cultured at 37℃ for 6 h. IPTG was added at a final concentration of 0, 0.5, 1 mmol/L and placed at 16℃ for 14 h to induce expression. Take the induced face bacterial solution and discard the supernatant by centrifugation at 4℃, add an appropriate amount of pure H_2_O for resuspension, and discard the supernatant by centrifugation again. The ratio of bacterial solution to the binding buffer is 5:1, adding an appropriate binding buffer to the precipitation for ultrasonic crushing. After sonication, the samples were centrifuged at 12,000 rpm for 30 min at 4℃, and the supernatant was collected and filtered on a 0.22 μm filter membrane and retained for protein purification.

LbCas12a protein was purified by nickel affinity chromatography. The protein was loaded on a nickel affinity chromatography column, the column was washed thoroughly with binding buffer to wash off the impurity protein, and the target protein was eluted with elution buffer. The protein was repurified and concentrated using an 8000–14,000 D dialysis bag and PEG20000.

### Screening and synthesis of gRNA and RAA primers

Based on multiple copies of IS6110 and IS1081 per MTB genome [16–18], we decided to use IS6110 and IS1081 as target sequences for MTB detection. This may be beneficial in improving assay sensitivity. MTB whole genome and MTB conserved sequences IS6110 and IS1081 were downloaded from the NCBI database *(*https://www.ncbi.nlm.nih.gov/gene*);* the accession number is: CP016972.1, IS6100 is located at 2,376,299–2,378,098; IS1081 is located at 2,841,720–2,842,968. According to the characteristics of LbCas12a protein that requires specific recognition of PAM sequences (TTN/TTTN) [19, 20], we designed and synthesized IS6110-gRNA1, IS6110-gRNA2, IS1081-gRNA1, and IS1081-gRNA2 (Table 1), while synthesizing IS6110 and IS1081 conserved sequence RAA primers (Table 2) [21, 22].

**Table 1:** ssDNA and gRNA sequences for detection of MTB DNA

component	ssDNA	gRNA
IS6110-gRNA1	CTATACAAGACCGAGCTGAT	UAAUUUCUACUAAGUGUAGAUAUCAGCUCGGUCUUGUAUAG
IS6110-gRNA2	GCGCGCTGGGTCGACTGGTT	UAAUUUCUACUAAGUGUAGAUAACCAGUCGACCCAGCGCGC
IS1081-gRNA1	GCCGGATGGAGCGCCTGGTC	UAAUUUCUACUAAGUGUAGAUGACCAGGCGCUCCAUCCGGC
IS1081-gRNA2	CGCTGCAGCAGCCAGTCCGG	UAAUUUCUACUAAGUGUAGAUCCGGACUGGCUGCUGCAGCG

**Table 2:** Primers for specific amplification of IS6110 and IS1081 fragments

component	ssDNA	reference
IS6110-RAA-F	GGTCGGAAGCTCCTATGACAATGCACTAGCC	[21]
IS6110-RAA-R	TTGAGCGTAGTAGGCAGCCTCGAGTTCGAC	
IS1081-RAA-F	CCAAGCTGCGCCAGGGCAGCTATTTCCCGGAC	[22]
IS1081-RAA-R	TTGGCCATGATCGACACTTGCGACTTGGA	

### RAA and CRISPR/Cas12a assay

RAA amplification reaction system: We added 25µL buffer A, 2µL forward primer, 2µL reverse primer (final concentration 0.4µmol/L), and 13.5µL ddH2O into a reaction tube containing lyophilized powder. Then we added 5µL DNA template and 2.5µL B buffer into the mixed reaction solution, mixed it well, and reacted at 39℃ for 30 min. The RAA amplification kit was purchased from Hangzhou Zhongce Biotechnology Co., LTD. (China).

Seven experimental groups were set up by reducing a component of the CRISPR/Cas12a detection system or adding mismatched gRNA and target DNA, and a control relationship was formed between groups 1–6 and 7, thereby verifying the integrity of the CRISPR/Cas12a detection system (Table S1). The CRISPR/Cas12a platform includes Cas12a, gRNA, fluorescent probe (ssDNA-FQ), H_2_O, and 10 × reaction buffer (10 mM Tris-HCl, 0.1 mM EDTA, 1 mM DTT, 100 mM NaCl), and 5 µL of the product amplified by RAA is made up to 20 µL and reacted on a Roche fluorescent quantitative PCR instrument at 37℃ for 30 min. In contrast, the fluorescent signal is detected every 1 min.

To construct a complete rapid detection platform and obtain the best detection effect, we optimized the concentrations of LbCas12a (50 nM, 100 nM, 200 nM, 400 nM), gRNA (50 nM, 100 nM, 200 nM, 1000 nM), ssDNA-FQ (100 nM, 200 nM, 500 nM, 800 nM) in each detection system.

### Evaluation of the specificity

We extracted the genomes of *Mycobacterium tuberculosis* H37Ra, *Mycobacterium Bovis* BCG, *Mycobacterium smegmatis*, *Pseudomonas aeruginosa*, *Klebsiella pneumoniae*, *Escherichia coli*, and *Staphylococcus aureus* by boiling cleavage method, respectively. We added seven genes with the same template volume to the RAA reaction system for isothermal amplification. After the reaction, the same book of amplified products was used for CRISPR/Cas12a detection to verify the specificity of primers and gRNA.

### Evaluation of the sensitivity

The concentration of the bacterial solution was determined according to an OD_600_ measurement of 1, equivalent to 3.13 × 10^7^ CFU/mL [23]. We performed 10-fold serial dilutions of H37 Ra from 3.13 × 10^7^ CFU/mL to 3.13 × 10^0^ CFU/mL in a gradient dilution, while ddH_2_O was used as a negative control; the test was repeated three times. The whole genome of H37 Ra was extracted by boiling lysis and utilized as an RAA amplification template. The eight groups of amplification products were detected by CRISPR/Cas12a system, and the fluorescence values were measured for sensitivity analysis.

### Selection and handling of clinical samples

To evaluate the efficacy of the TB-CRISPR system on clinical specimen detection, we selected 504 patients with suspected *mycobacterium tuberculosis* infection who were admitted to the Second People’s Hospital of Weifang City from May 25, 2022, to June 30, 2022. The 504 clinical samples included 217 sputa, 194 bronchoalveolar lavage fluid (BALF), 54 hydrothorax, 17 tissues, 10 urine, 4 puncture fluid, 3 cerebrospinal fluid, 2 ascites, 2 pus, and 1 lung puncture tissue. All clinical samples were simultaneously preprocessed and tested for BACTEC 960 culture and CRISPR.

**Fig. 1:**
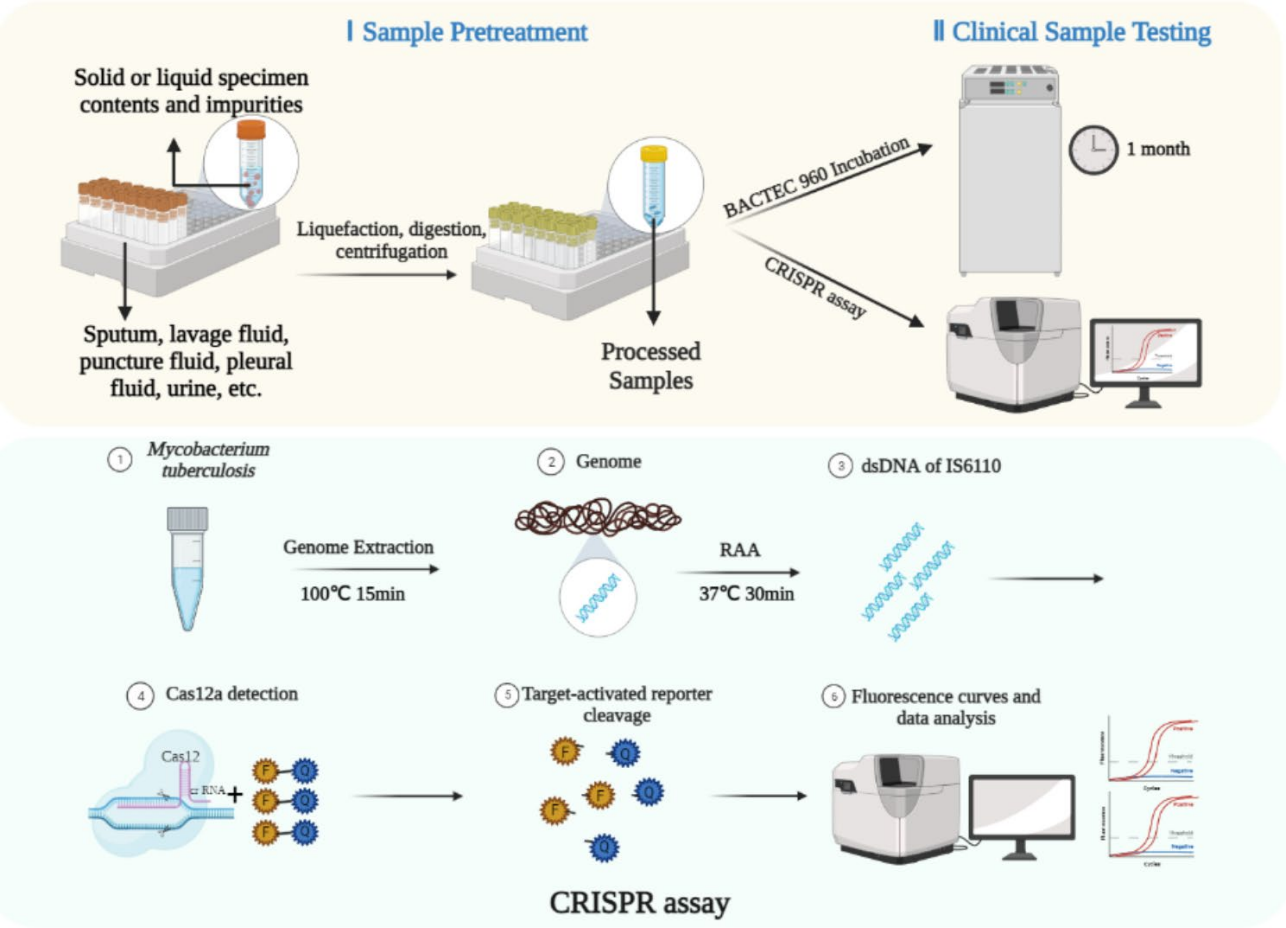
Scheme of TB-CRISPR

Preparation of 2% NALC-NaOH specimen pretreatment solution: 100 mL mixture was prepared with 50 mL of 4% NaOH solution, and 50 mL of 29% sodium citrate solution, 0.5 g of NALC was added and mixed well. Use within 24 h. Pick up about 5 mL (not more than 10 mL) of sputum into a 50 mL labeled centrifuge tube, add the same amount of 2% NALC-NaOH pretreatment solution (not more than 10 mL), and vigorously vortex for 20 s. If the sputum is viscous, add more pretreatment solution and repeat shaking; stand at room temperature for 15 to 20 min; do not exceed 20 min (to prevent killing tuberculosis); add sterile PBS (pH 6.8) to about 50 mL and cap tightly; centrifuge 3000 g for 15 min; discard the upper night; add 1 to 3 ml PBS to neutralize pH to 6.8. We aspirated 2 mL of treated specimens and extracted nucleic acids by heat lysis for TB-CRISPR system detection, and the remaining samples were cultured with BACTEC960 to obtain culture results in one month later (Fig. 1).

### Data analysis

For each sample, each experiment was repeated at least three times, and all experimental results were expressed as the mean of replicate tests. Statistical significance is indicated at P-value less than *P < 0.1; **P < 0.05; and ***P < 0.01.

Statistical analysis and plotting, including Limit of detection (Lod), receiver operating characteristic (ROC), the area under the curve (AUC), specificity, sensitivity, positive likelihood ratio (PLR), negative likelihood ratio (NLR), positive predictive value (PPV), negative predictive value (NPV), and 95% confidence interval (95% CI) were performed using IBM SPSS Statistics 26.0, GraphPad Prism 8.0.2, and report ROC package for R.

## Results

### Validation of cleavage activity of LbCas12a protein

The theoretical molecular weight of LbCas12a protein was 143 kDa, and a band of about 143 kDa was expressed by the strain containing the recombinant plasmid under the action of the inducer IPTG by induction. The band size was consistent with the theoretical molecular weight of LbCas12a protein, indicating that LbCas12a protein was successfully expressed in this study. By adjusting different IPTG concentrations, it was found that the target protein expression was highest when the induction temperature was at 16 °C, and the final IPTG concentration was 1 mmol/L. To obtain a single LbCas12a protein with higher concentration, the imidazole concentration in Binding Buffer and Elution Buffer was optimized, and it was found that the protein purification effect was the best when the imidazole concentration in the binding buffer was 20 mmol/L, and the imidazole concentration in elution buffer was 250 mmol/L.

LbCas12a protein binding to protospacer adjacent motif directly leads to local melting of PAM downstream target sequence DNA, causing downstream sequence complementation with specifically recognized gRNA, thereby activating cleavage of target DNA by Cas12a protein [24]. The activity of the Cas12a protein directly affects the detection efficacy of the CRISPR/Cas12a system. We chose to amplify the IS1081 (187 bp) conserved sequence for validation. When the RAA amplification product was added to the CRISPR/Cas12a system, the complex of IS1081-gRNA1 and Cas12a protein could cleave target DNA in cis, resulting in a decrease in the number of IS1081-DNA (Figure S1).

### CRISPR/Cas12a Integrity Verification

Different experimental groups were set up by lacking a component in the detection system or adding mismatched gRNA and target DNA, and three independent replicates were performed for each group to detect fluorescence values. When each element in the detection system is correct and complete, it can produce significant fluorescence signals. Experimental groups with missing and mismatched components could not specifically identify and produce fluorescence signals (Figure S2).

### Optimization of CRISPR/Cas12a assay

#### Selection of gRNA

**Fig. 2:**
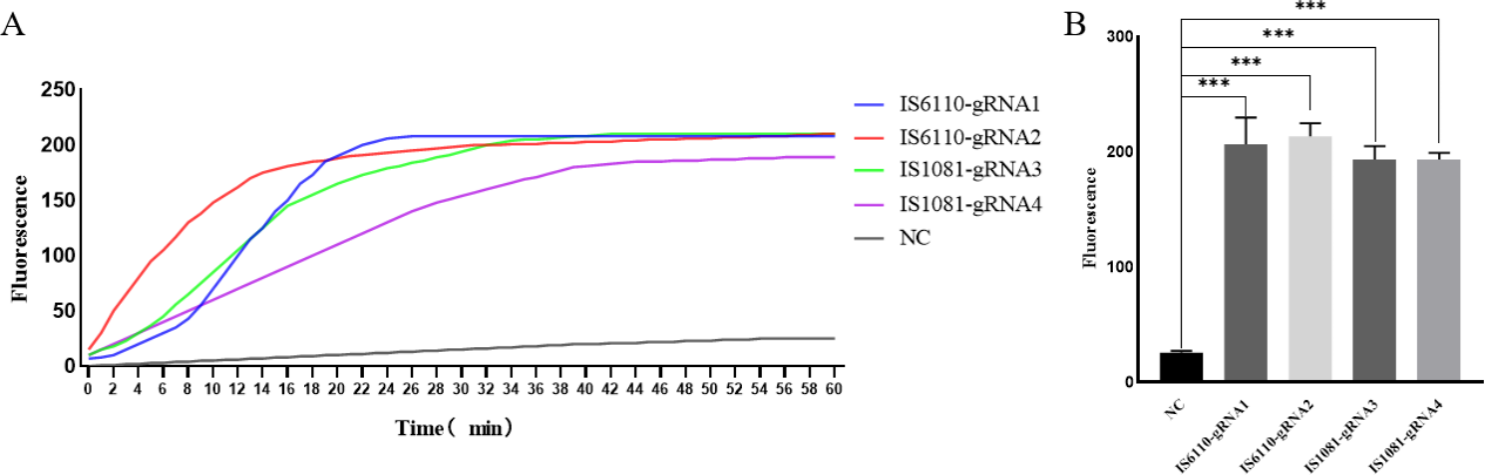
Comparison of different gRNA detection capabilities (**A**) fluorescence curve; Blue:IS6110-gRNA1; Red: IS6110-gRNA2; Green: IS1081-gRNA1; Purple: IS1081-gRNA2; Gray: negative control; (**B**) Fluorescence value of four gRNAs NC: negative control; ***: P < 0.001

After analysis of MTB conserved sequences IS6110 and IS1081, four different gRNAs (IS6110-gRNA1, IS6110-gRNA2, IS1081-gRNA1, IS1081-gRNA2) were added to the CRISPR/Cas12a system for validation (Fig. 2). To ensure that the four groups of experiments could complete the reaction, we chose the time point (60 min) when all four gRNA could reach the plateau as the detection endpoint. According to the test results, IS6110-gRNA2 has the highest fluorescence value and the fastest speed and can get the plateau in 20 min. Therefore, we chose IS6110-gRNA2 for the follow-up study. During the fluorescence detection process, the four groups of gRNAs had the same components, concentration, and detection time, and the fluorescence intensity of gRNA was determined by the fluorescence value of the detection endpoint.

#### Establishment of CRISPR/Cas12a assay

Assays were performed in 10 × reaction buffer, 200 nM purified LbCas12a protein, 500 nM ssDNA-FQ, 50 nM gRNA, and 5 µL of DNA in a reaction volume of 20 µL. Real-time monitoring of the fluorescence signal was performed using a Roche Light Cycler 480 fluorescence quantifier with an excitation wavelength of 485 nm, an emission wavelength of 535 nm, and a temperature of 37 °C, and the fluorescence signal was detected every 1 min for 30 cycles.

### CRISPR/Cas12a specificity analysis

**Fig. 3:**
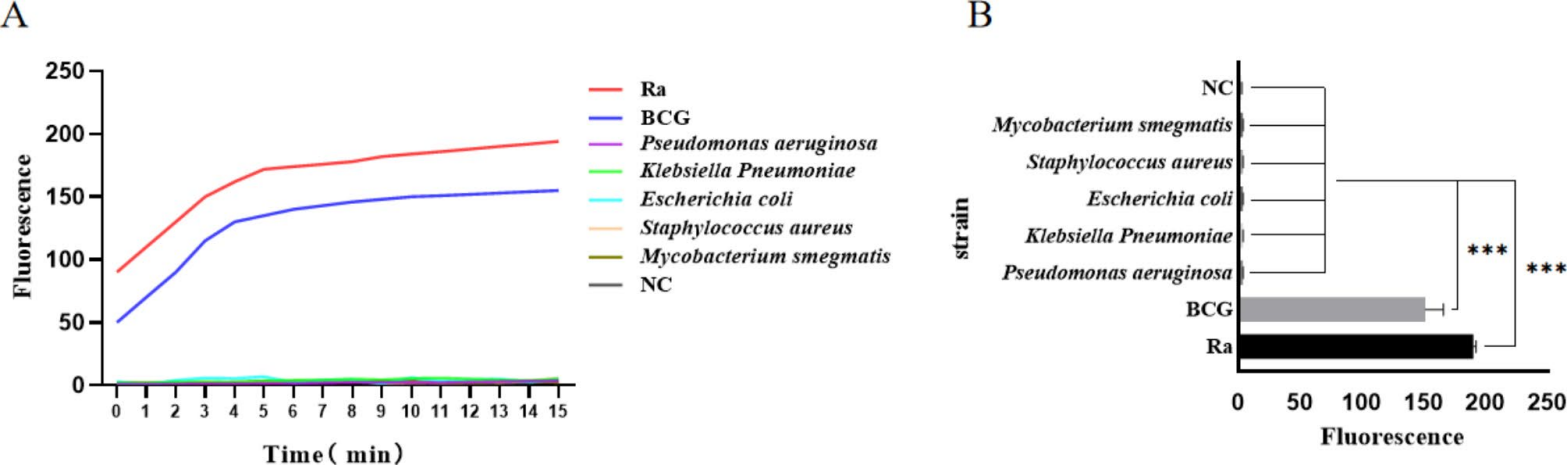
Specificity test based on CRISPR system (**A**) CRISPR detection curves for the specificity of seven different strains; (**B**) column chart of CRISPR detection of specificity of seven different strains NC: negative control; ***: P < 0.001

When the IS6110 gene fragment was present in the CRISPR/Cas12a detection system, a higher fluorescence value could be detected, and the curve amplification characteristics were evident, which showed that the fluorescence value reached a plateau after increasing. The fluorescence value tended to be stable. We verified the specificity of the TB-CRISPR detection system by using eight different strains of *Mycobacterium tuberculosis* H37Ra, *Mycobacterium Bovis* BCG, *Mycobacterium smegmatis*, *Pseudomonas aeruginosa*, *Klebsiella pneumoniae*, *Escherichia coli*, and *Staphylococcus aureus*. As shown in Fig. 3, H37Ra and BCG detected high fluorescence values due to IS6110 conserved sequences, and the other five strains and negative control could not detect statistically significant fluorescence values. When the reaction lasted for 15 min, both H37Ra and BCG could detect strong fluorescence signal values and showed statistically significant differences from the other strains, so we stopped the detection at 15 min.

### CRISPR/Cas12a sensitivity analysis

**Fig. 4:**
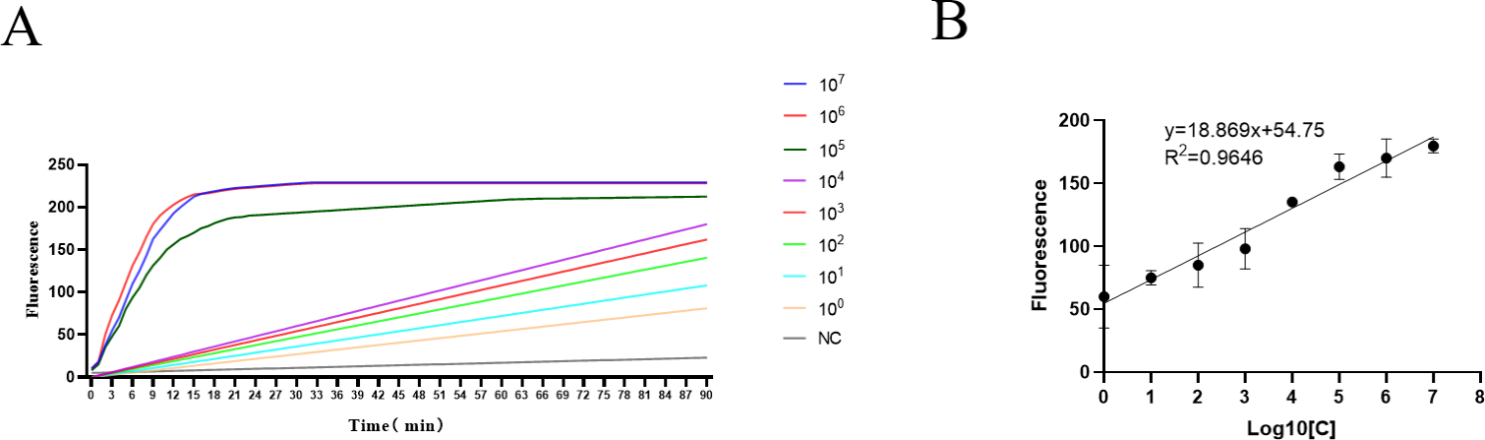
Sensitivity test based on CRISPR system (**A**) Real-time fluorescence intensity alteration of H37 Ra assay for different concentrations of targets; (**B**) The calibration plots of fluorescence intensity versus the logarithm of the target concentration. NC: negative control

Sensitivity was determined using serially diluted H37Ra from 3.13 × 10^0^ CFU/mL to 3.13 × 10^7^ CFU/mL. The results showed that with the decrease in bacterial concentration, the fluorescence value produced by LbCas12a after cutting target DNA gradually decreased, and the cutting rate gradually decreased. A strong fluorescence signal could still be detected when the bacterial concentration was 3.13 CFU/mL (Fig. 4A), with a significant signal difference compared with the negative control group. Therefore, the Lod of CRISPR/Cas12a was 3.13 CFU/mL H37Ra. A standard curve plotting fluorescence values versus bacterial solution concentration was also obtained by measuring fluorescence values as y = 1.869x + 54.75 (R^2^ = 0.9646) (Fig. 4B).

### Clinical validation

**Fig. 5:**
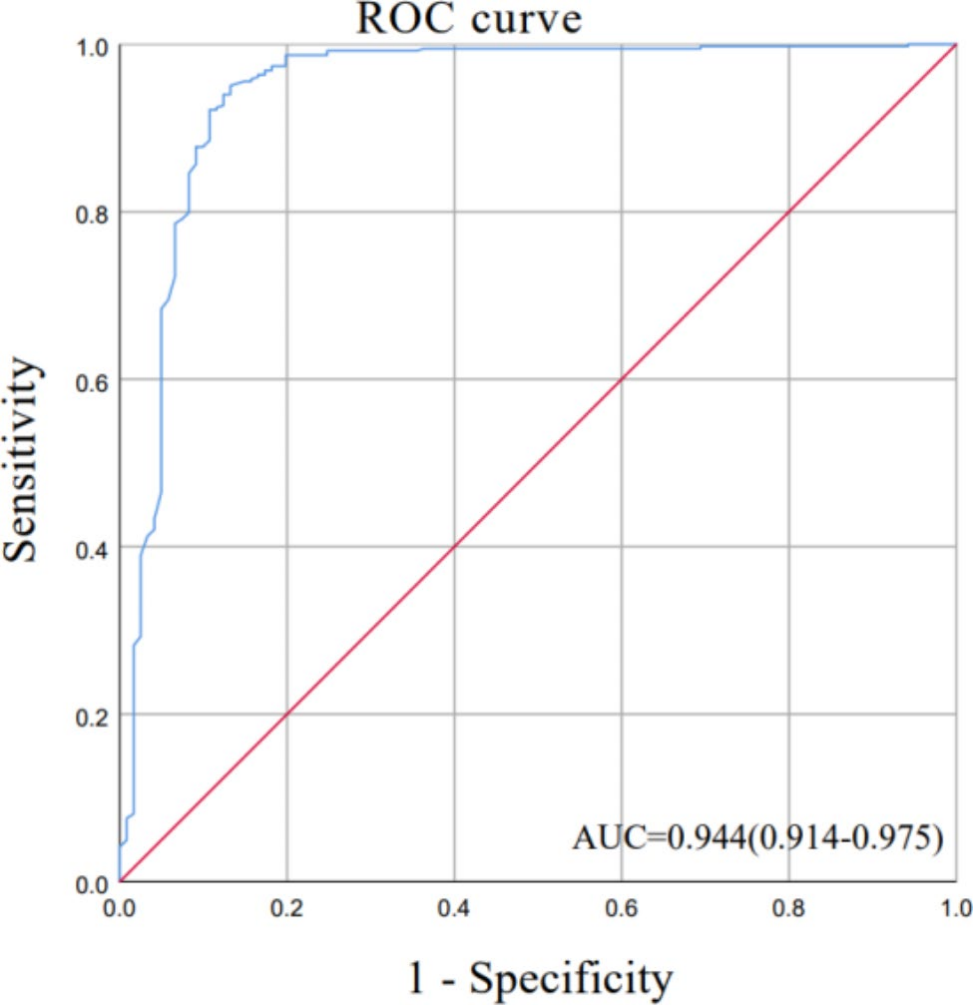
ROC curve 504 ROC Curve Analysis for Relative Fluorescence Values of Clinical Samples

**Fig. 6:**
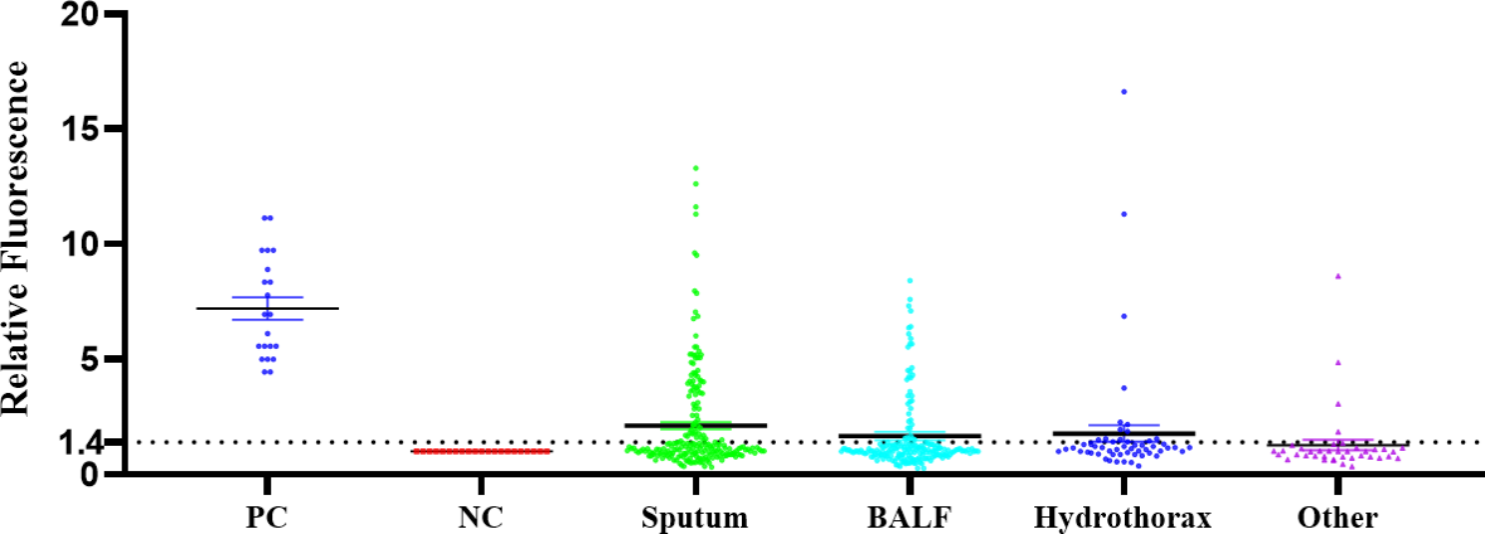
Relative fluorescence values for clinical samples. Samples included 217 sputa, 194 bronchoalveolar lavage fluid (BALF), 54 hydrothorax, 39 other (17 tissues, 10 urine, 4 puncture fluid, 3 cerebrospinal fluid, 2 ascites, 2 pus, and 1 lung puncture tissue)

To evaluate the clinical application effect of TB-CRISPR, 504 suspected tuberculosis samples were tested. All samples were analyzed in 21 independent batches. H37Ra and sterile enzyme-free H_2_O were positive (PC) and negative (NC) controls in each assay. NC signals were used to normalize other sample signals. The relative fluorescence values of all samples were summarized and analyzed in ROC curves (Fig. 5), and it was finally determined that samples with a signal 1.4 times higher than the negative fluorescence value were considered positive. In contrast, samples below this cut-off value were considered negative. Eventually, we identified 129 positive and 375 negative samples for TB-CRISPR testing (Fig. 6). Using the BACTEC960 culture results (120 positives and 384 negatives) as the gold standard. The sensitivity and specificity of the TB-CRISPR detection system were calculated to be 0.883 (0.809–0.932) and 0.940 (0.910–0.961), respectively (Table 3). According to ROC curve analysis, AUC was 0.944 (0.914–0.975) (Fig. 5). Additional key performance characteristics for TB-CRISPR assay analysis in clinical samples were subsequently calculated. The positive likelihood ratio (PLR) was 14.747 (9.870-22.035), and the negative likelihood ratio (NLR) was 0.124 (0.076–0.203). The positive predictive value (PPV) was 0.822 (0.742–0.881), and the negative predictive value (NPV) was 0.963 (0.937–0.979).

**Table 3:** Clinical Diagnostic Performance of CRISPR-MTB Assay on Clinical Samples

Reference method	Results of the reference method	TB-CRISPR		Total
		Positive	Negative	
BACTEC 960	Positive	106	14	120
	Negative	23	361	384
Total		129	375	504
Specificity		0.940 (0.910–0.961)		
Sensitivity		0.883 (0.809–0.932)		

**Fig. 7:**
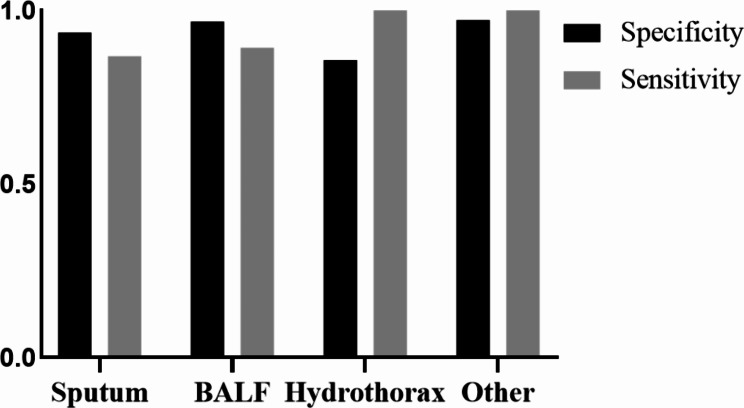
Specificity and sensitivity by clinical sample type

We compared the TB-CRISPR in different sample types to further analyze the diagnostic performance. The diagnostic performance varied across sample types (Fig. 7, Table S2). Among 504 specimens, BALF had the highest accuracy with a specificity of 0.968 (152/157) and sensitivity of 0.982 (33/37), slightly higher than sputum specimens with the largest number of samples with a specificity of 0.936 (132/141) and a sensitivity of 0.868 (66/76). As the tissue with the fourth largest number of clinical sample types (17 cases), its specificity and sensitivity were both 1, which was the best-performing sample type among the test results. It is worth noting that the sensitivity of pleural fluid and lung puncture tissue is 1; the specificity of puncture fluid, cerebrospinal fluid, and ascites is 1; while the specificity of Pus is only 0.5, and there is a phenomenon of very high or very low specificity or sensitivity. Because the number of samples in the above types is small and the results may be biased, we classified various sample types with less than 50 clinical samples into the “Other” group for unified analysis.

## Discussion

Improving the sensitivity of MTB detection is an essential prerequisite for early diagnosis and treatment of TB. Smear acid-fast staining is one of the routine methods for bacteriological detection because it is simple and rapid. However, the detection rate of positive samples by this method is low, and 591,000 bacteria per milliliter of samples are generally required to show positive results, which are related to the degree of disease, the intensity of infectivity, and the quality of sputum samples, so this method has low sensitivity and high missed diagnosis rate [21]. The culture method is time-consuming, clinicians cannot obtain laboratory results to determine drug-resistant TB, individualized anti-TB treatment regimens cannot be developed, and previous empirical anti-TB regimens are ineffective in controlling tuberculosis. In this study, a rapid detection technique for MTB was established by RAA isothermal amplification combined with CRISPR/Cas system with a detection limit as low as 3.13 CFU/mL, approximately 53 copies/mL. From the experimental data of this study, it can be seen that TB-CRISPR detected 504 patients with suspected TB, with a specificity of 0.946 and a sensitivity of 0.883, and can make a diagnosis of patients with suspected TB within 1.5 h, with high sensitivity and specificity, which largely compensates for the lack of traditional diagnostic methods.

Although TB-CRISPR still uses the more complex instrument of LigheCycler 480 for detection, we can simplify the CRISPR technology detection method in the future [25, 26]. Such as combining lateral flow chromatography, completing the detection reaction with only a simple constant temperature instrument, visually displaying the detection results on the test strip, and realizing visual detection; at the same time, CRISPR can also be combined with smartphones and smartphone camera acts as a “microscope”, which can detect fluorescence and report the results, facilitating the promotion of universal home detection, and maintaining patient privacy. TB-CRISPR has broad development prospects, and this technology is more suitable for application and promotion in resource-poor areas and grass-roots laboratories.

Compared with Xpert MTB/RIF, loop-mediated isothermal amplification (LAMP), and Simultaneous amplification and testing (SAT), TB-CRISPR took only 90 min on average, including 20 min for rapid DNA extraction, 30 min for RAA amplification, and 30 min for Cas12a detection, while the most commonly used Xpert MTB/RIF in clinical practice took 120 min to report the results. The gold standard culture method took one month to report the negative bacteria, and TB-CRISPR showed significant advantages in the detection time [27]. At the same time, some research teams point out that the price of CRISPR detection of pathogens is about half of the price of PCR detection [28], and the above aspects suggest that this technology has greater advantages for the early diagnosis of MTB and provides strong technical support for subsequent tuberculosis diagnosis and prevention and control.

Both selections of pathogen targets and the processing of samples have some impact on the detection of CRISPR systems. Four gRNAs designed for IS6110 and IS1081 were compared longitudinally based on fluorescence profiles. Under the same concentration of the bacterial solution, H37Ra contains 17 copies of IS6110 and only 5–7 copies of IS1081 [17, 29]. Therefore, IS6110 reached the fluorescence platform faster than IS1081 after RAA isothermal amplification and could detect as low as 53 copies /mL, indicating that CRISPR/Cas12a is an ultra-sensitive detection technology. Because we used a relatively simple and fast nucleic acid extraction method to better adapt to resource-poor areas, which may lead to insufficient nucleic acid extraction quality, 14 positive clinical samples did not detect sufficiently high fluorescence signals during testing. However, it may also be a false negative caused by mutations in the gRNA recognition sequence, and we can improve the TB-CRISPR system to increase multiplex target detection (IS6110, IS1081, 16s rRNA, gyrB, etc.), thereby avoiding the generation of false negatives and also improving detection sensitivity.

TB-CRISPR technology has high sensitivity and good diagnostic efficiency. However, there are still some limitations: (1) There are many influencing factors, poor repeatability of CRISPR, and the amplification time of RAA will also affect the sensitivity of the entire detection system, so it is not possible to accurately quantify the concentration of bacterial solution at present; (2) In this study, BACTEC960 culture method was selected as the gold standard, but the positive rate of traditional culture method was not high. The diagnostic results would be more convincing if the reference standard were based on the final clinical diagnosis. However, in this study, a relatively simple pyrolysis method was chosen to extract the genome, so the time of the whole detection process was shortened. Genome extraction and testing costs about $6.90, which is less expensive than other molecular diagnostic methods. At the same time, 504 clinical samples were included in this study, with a large sample size and multiple sample types, and the diagnostic results were more representative. Zhang’s team can diagnose TB by detecting *Mycobacterium tuberculosis* cell-free DNA in blood and assessing TB treatment response, providing a new way of thinking for non-sputum-based TB diagnosis [30].

Various pathogen detection platforms based on CRISPR technology currently require an initial amplification process, such as using LAMP, RPA, RAA, and other amplification of pathogen target sequences, thereby improving the sensitivity of the entire detection system. However, introducing the amplification step prolongs the detection time and increases the complexity of the operation, raising the risk of carryover contamination resulting in false positives in the detection. In addition, it does not fully use the original ultrasensitive and strict PAM recognition characteristics of CRISPR and reduces the actual advantages of the CRISPR system. At present, amplification-free CRISPR technology has been used to detect a novel coronavirus, and its sensitivity can reach 30 copies/mL [31], with good detection results. Hence, amplification-free CRISPR technology in MTB detection may be a key technology for future diagnostics.

## Conclusions

In this study, CRISPR-Cas12a was combined with the RAA amplification technique to establish a new method for detecting MTB, TB-CRISPR, which showed high diagnostic value through the validation of 504 clinical samples and enriched the molecular diagnostic methods of tuberculosis. At the same time, it reduces the use of expensive thermal cyclers, shortens the detection cycle, and provides an economical, efficient, and simple platform for grassroots laboratories and field detection. Establishing TB-CRISPR lays a foundation for the early diagnosis of tuberculosis and point-of-care testing.

## Patents

There is a patent for “A composition, kit and its application for the detection of *Mycobacterium tuberculosis*,” which is under the application.

### Electronic supplementary material

Below is the link to the electronic supplementary material.

Supplementary Material 1

Supplementary Material 2

## Data Availability

All data of the article has been published, and The login link is: 10.57760/sciencedb.07522. The primary data in this study can be obtained from the survey itself without any sequencing work involved. Other data can be obtained by contacting the corresponding authors.
