# Supplementary material for: A new method for the detection of Mycobacterium tuberculosis based on the CRISPR/Cas system
Source: BMC Infect Dis. 2023 Oct 11;23:680. doi: 10.1186/s12879-023-08656-4 (PMC10568934; doi:10.1186/s12879-023-08656-4)
Supplement: Supplementary file 1 — Supplementary Material 1 [file 12879_2023_8656_MOESM1_ESM.docx]

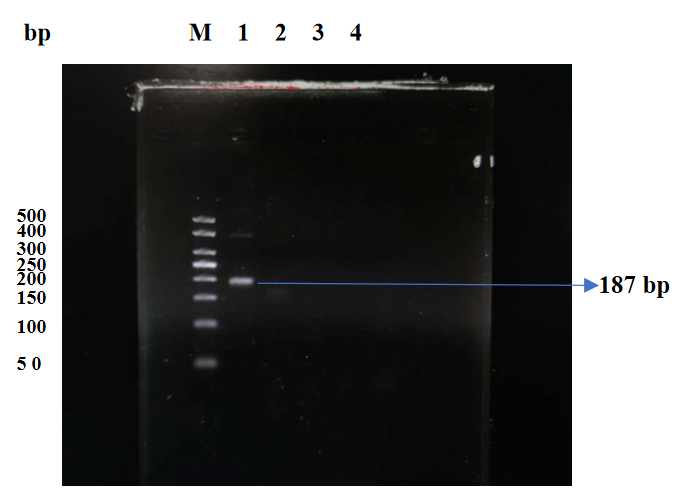


**Figure S1 Cas12a Cutting Activity Verification** M: Marker；1: The system does not contain Cas12a protein；2-4: Three independent replicates containing Cas12a protein in the system.


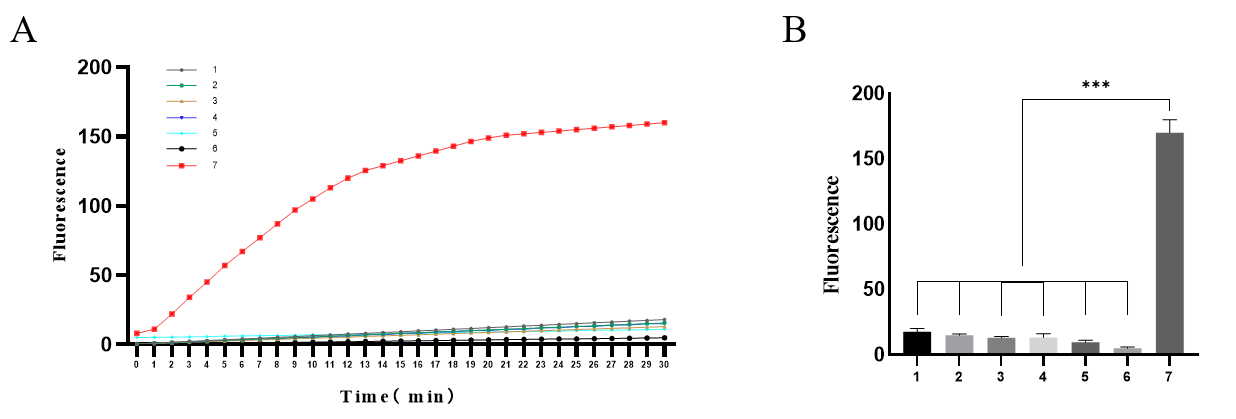


**Figure S2 Integrity verification of CRISPR/Cas12a detection system** **(A)** Fluorescence curve1: LbCas12a+ target+ FQ; 2: gRNA+ target+ FQ; 3: LbCas12a+ target+ gRNA; 4: LbCas12a+ gRNA + FQ; 5: LbCas12a+ target(IS6110)+ FQ+ gRNA(IS1081-gRNA2); 6: LbCas12a+ target(IS1081)+ FQ+ gRNA(IS6110-gRNA2); 7: LbCas12a+ target(IS6110)+ FQ+ gRNA(IS6110-gRNA2); **(B)** Histogram of fluorescence value ;***: P<0.001.

**Table S1 Integrity verification of CRISPR/Cas12a detection system**

| Component | 1 | 2 | 3 | 4 | 5 | 6 | 7 |
| --- | --- | --- | --- | --- | --- | --- | --- |
| LbCas12a | **+** | **-** | **+** | **+** | **+** | **+** | **+** |
| gRNA（IS6110-1） | **-** | **+** | **+** | **+** | **-** | **+** | **+** |
| gRNA（IS1081-1） | **-** | **-** | **-** | **-** | **+** | **-** | **-** |
| Target DNA（IS6110） | **+** | **+** | **+** | **-** | **+** | **-** | **+** |
| Target DNA（IS1081） | **-** | **-** | **-** | **-** | **-** | **+** | **-** |
| ssDNA-FQ | **+** | **+** | **-** | **+** | **+** | **+** | **+** |
| Buffer | **+** | **+** | **+** | **+** | **+** | **+** | **+** |
| H_2_O | **+** | **+** | **+** | **+** | **+** | **+** | **+** |

Note: +: add this component; -: lack of this component

**Table S2 Specificity and Sensitivity by Sample Type**

| Type | BACTEC 960 | TB-CRISPR assay | | | Total | Specificity | Sensitivity |
| --- | --- | --- | --- | --- | --- | --- | --- |
|  |  | Positive | Negative | |  |  |  |
| Sputum | Positive | 66 | 10 | | 217 | 0.936 | 0.868 |
|  | Negative | 9 | 132 | |  |  |  |
| Bronchoalveolar lavage fluid | Positive | 33 | 4 | | 194 | 0.968 | 0.892 |
|  | Negative | 5 | | 152 |  |  |  |
| Hydrothorax | Positive | 5 | | 0 | 54 | 0.857 | 1 |
|  | Negative | 7 | | 42 |  |  |  |
| Tissue | Positive | 1 | | 0 | 17 | 1 | 1 |
|  | Negative | 0 | | 16 |  |  |  |
| Urine | Positive | 1 | | 0 | 10 | 0.9 | - |
|  | Negative | 0 | | 9 |  |  |  |
| Puncture fluid | Positive | 0 | | 0 | 4 | 1 | - |
|  | Negative | 0 | | 4 |  |  |  |
| Cerebrospinal fluid | Positive | 0 | | 0 | 3 | 1 | - |
|  | Negative | 0 | | 3 |  |  |  |
| Ascites | Positive | 0 | | 0 | 2 | 1 | - |
|  | Negative | 0 | | 2 |  |  |  |
| Pus | Positive | 0 | | 0 | 2 | 0.5 | - |
|  | Negative | 1 | | 1 |  |  |  |
| Lung puncture tissue | Positive | 1 | | 0 | 1 | - | 1 |
|  | Negative | 0 | | 0 |  |  |  |
| Other  (Containing Tissue, Urine, Puncture fluid, Cerebrospinal fluid, Ascites, Pus) | Positive | 3 | | 0 | 39 | 0.972 | 1 |
|  | Negative | 1 | | 35 |  |  |  |
