# Supplementary material for: A new method for the detection of Mycobacterium tuberculosis based on the CRISPR/Cas system
Source: BMC Infect Dis. 2023 Oct 11;23:680. doi: 10.1186/s12879-023-08656-4 (PMC10568934; doi:10.1186/s12879-023-08656-4)
Supplement: Supplementary file 2 — Supplementary Material 2 [file 12879_2023_8656_MOESM2_ESM.docx]

Table S3 Clinical Sample Fluorescence Values

| Patients | Type | BACTEC 960 | TB-CRISPR | Relative fluorescence value |
| --- | --- | --- | --- | --- |
| 1 | sputum | - | - | 0.87 |
| 2 | sputum | - | - | 0.73 |
| 3 | sputum | + | - | 1.13 |
| 4 | sputum | + | + | 1.78 |
| 5 | sputum | + | + | 4.07 |
| 6 | sputum | + | + | 9.53 |
| 7 | BALF | + | + | 3.20 |
| 8 | sputum | - | - | 0.69 |
| 9 | sputum | + | + | 4.87 |
| 10 | sputum | - | - | 1.36 |
| 11 | sputum | - | - | 0.96 |
| 12 | sputum | - | - | 0.96 |
| 13 | sputum | - | - | 1.13 |
| 14 | hydrothorax | + | + | 3.76 |
| 15 | BALF | - | - | 0.96 |
| 16 | BALF | - | - | 0.96 |
| 17 | BALF | - | - | 1.00 |
| 18 | tissues | - | - | 1.04 |
| 19 | sputum | - | - | 0.96 |
| 20 | sputum | - | - | 0.82 |
| 21 | sputum | + | + | 5.20 |
| 22 | sputum | + | + | 4.11 |
| 23 | hydrothorax | - | - | 1.22 |
| 24 | sputum | + | + | 3.53 |
| 25 | sputum | + | + | 3.48 |
| 26 | sputum | + | + | 4.32 |
| 27 | sputum | - | - | 0.93 |
| 28 | sputum | + | + | 3.82 |
| 29 | BALF | - | - | 1.18 |
| 30 | BALF | - | - | 1.05 |
| 31 | BALF | - | - | 0.95 |
| 32 | BALF | - | - | 0.72 |
| 33 | BALF | - | - | 0.85 |
| 34 | BALF | - | - | 0.95 |
| 35 | sputum | + | + | 4.82 |
| 36 | BALF | - | - | 1.02 |
| 37 | BALF | + | + | 4.13 |
| 38 | BALF | - | - | 1.15 |
| 39 | BALF | + | + | 4.48 |
| 40 | hydrothorax | - | - | 1.02 |
| 41 | hydrothorax | - | - | 1.18 |
| 42 | hydrothorax | - | - | 1.22 |
| 43 | sputum | + | + | 3.85 |
| 44 | sputum | + | + | 5.07 |
| 45 | sputum | - | - | 1.45 |
| 46 | sputum | - | - | 1.40 |
| 47 | BALF | - | - | 1.07 |
| 48 | sputum | - | - | 0.90 |
| 49 | sputum | - | + | 1.61 |
| 50 | sputum | - | - | 1.06 |
| 51 | sputum | - | - | 1.13 |
| 52 | sputum | + | + | 6.02 |
| 53 | sputum | - | - | 0.91 |
| 54 | sputum | - | - | 1.02 |
| 55 | sputum | + | + | 5.17 |
| 56 | sputum | - | - | 0.96 |
| 57 | sputum | - | - | 1.09 |
| 58 | sputum | + | + | 3.13 |
| 59 | hydrothorax | - | - | 1.30 |
| 60 | tissues | - | - | 1.15 |
| 61 | BALF | - | - | 0.93 |
| 62 | BALF | - | - | 0.69 |
| 63 | BALF | + | + | 5.91 |
| 64 | BALF | - | - | 0.28 |
| 65 | hydrothorax | - | - | 1.02 |
| 66 | BALF | - | + | 1.57 |
| 67 | BALF | - | + | 1.69 |
| 68 | BALF | - | - | 0.48 |
| 69 | BALF | - | - | 1.13 |
| 70 | BALF | - | - | 0.87 |
| 71 | puncture fluid | - | - | 0.80 |
| 72 | sputum | + | + | 4.06 |
| 73 | sputum | + | + | 4.53 |
| 74 | sputum | + | + | 4.02 |
| 75 | BALF | - | - | 1.15 |
| 76 | BALF | - | - | 1.18 |
| 77 | BALF | - | - | 0.82 |
| 78 | BALF | - | - | 0.72 |
| 79 | BALF | - | - | 0.83 |
| 80 | BALF | - | - | 0.95 |
| 81 | sputum | + | + | 2.57 |
| 82 | sputum | + | + | 2.05 |
| 83 | sputum | - | - | 1.25 |
| 84 | sputum | + | + | 1.55 |
| 85 | sputum | - | - | 1.25 |
| 86 | sputum | + | - | 1.28 |
| 87 | sputum | - | - | 1.32 |
| 88 | sputum | - | - | 1.15 |
| 89 | sputum | + | + | 3.78 |
| 90 | hydrothorax | - | - | 1.28 |
| 91 | hydrothorax | - | + | 1.55 |
| 92 | hydrothorax | - | + | 1.50 |
| 93 | hydrothorax | - | - | 1.12 |
| 94 | hydrothorax | - | - | 1.28 |
| 95 | hydrothorax | - | - | 0.55 |
| 96 | BALF | - | - | 0.77 |
| 97 | BALF | - | - | 1.12 |
| 98 | BALF | - | - | 1.01 |
| 99 | BALF | - | - | 1.13 |
| 100 | sputum | - | - | 1.21 |
| 101 | sputum | - | - | 1.10 |
| 102 | sputum | + | - | 1.31 |
| 103 | sputum | + | - | 0.94 |
| 104 | sputum | - | - | 1.12 |
| 105 | sputum | - | - | 1.01 |
| 106 | sputum | - | - | 1.04 |
| 107 | sputum | + | + | 3.68 |
| 108 | sputum | - | - | 1.11 |
| 109 | sputum | - | - | 1.00 |
| 110 | sputum | - | - | 0.82 |
| 111 | sputum | - | - | 0.70 |
| 112 | sputum | + | + | 1.81 |
| 113 | sputum | - | - | 0.95 |
| 114 | sputum | - | - | 1.01 |
| 115 | sputum | + | + | 1.65 |
| 116 | BALF | - | - | 1.23 |
| 117 | BALF | + | + | 1.55 |
| 118 | BALF | - | - | 0.81 |
| 119 | BALF | - | - | 1.10 |
| 120 | BALF | - | - | 1.04 |
| 121 | BALF | - | - | 0.93 |
| 122 | BALF | - | - | 0.67 |
| 123 | BALF | + | - | 1.38 |
| 124 | BALF | - | - | 0.98 |
| 125 | sputum | + | + | 1.71 |
| 126 | BALF | - | - | 1.02 |
| 127 | BALF | - | - | 0.96 |
| 128 | BALF | + | + | 6.42 |
| 129 | BALF | - | - | 1.09 |
| 130 | hydrothorax | - | - | 1.00 |
| 131 | sputum | + | + | 5.36 |
| 132 | sputum | - | - | 0.60 |
| 133 | sputum | - | - | 0.87 |
| 134 | sputum | + | + | 5.22 |
| 135 | BALF | + | + | 4.24 |
| 136 | BALF | - | - | 1.07 |
| 137 | BALF | - | - | 0.87 |
| 138 | BALF | - | - | 0.93 |
| 139 | BALF | - | - | 0.42 |
| 140 | BALF | + | + | 3.42 |
| 141 | BALF | - | - | 0.51 |
| 142 | hydrothorax | - | - | 1.09 |
| 143 | sputum | - | - | 0.87 |
| 144 | sputum | - | - | 1.36 |
| 145 | sputum | + | + | 3.41 |
| 146 | sputum | - | - | 1.24 |
| 147 | sputum | - | - | 1.13 |
| 148 | sputum | - | - | 1.02 |
| 149 | sputum | - | - | 1.00 |
| 150 | sputum | + | + | 3.96 |
| 151 | hydrothorax | - | - | 0.94 |
| 152 | BALF | - | - | 1.02 |
| 153 | BALF | - | - | 0.72 |
| 154 | BALF | - | - | 0.50 |
| 155 | BALF | + | + | 1.56 |
| 156 | BALF | - | - | 0.85 |
| 157 | BALF | + | + | 2.30 |
| 158 | sputum | + | + | 2.28 |
| 159 | sputum | - | - | 0.35 |
| 160 | sputum | - | - | 1.00 |
| 161 | hydrothorax | - | - | 1.00 |
| 162 | hydrothorax | - | - | 0.96 |
| 163 | hydrothorax | + | + | 2.28 |
| 164 | sputum | + | + | 2.57 |
| 165 | sputum | - | - | 0.87 |
| 166 | sputum | + | + | 2.39 |
| 167 | sputum | + | + | 4.06 |
| 168 | sputum | - | - | 1.02 |
| 169 | sputum | - | - | 0.93 |
| 170 | sputum | + | + | 2.97 |
| 171 | sputum | + | + | 2.14 |
| 172 | sputum | - | - | 0.93 |
| 173 | hydrothorax | - | - | 0.88 |
| 174 | ascites | - | - | 0.79 |
| 175 | sputum | - | - | 0.96 |
| 176 | sputum | + | + | 5.54 |
| 177 | sputum | - | - | 0.75 |
| 178 | tissues | - | - | 1.01 |
| 179 | tissues | - | - | 1.13 |
| 180 | BALF | + | + | 5.68 |
| 181 | BALF | - | - | 1.01 |
| 182 | BALF | + | + | 6.38 |
| 183 | BALF | - | - | 1.01 |
| 184 | BALF | - | - | 0.96 |
| 185 | BALF | - | - | 1.06 |
| 186 | BALF | - | - | 1.01 |
| 187 | BALF | - | - | 1.26 |
| 188 | BALF | - | - | 1.18 |
| 189 | BALF | - | - | 1.29 |
| 190 | BALF | - | - | 1.13 |
| 191 | BALF | + | + | 5.68 |
| 192 | BALF | - | - | 1.21 |
| 193 | BALF | - | - | 1.13 |
| 194 | BALF | - | - | 1.37 |
| 195 | BALF | - | - | 0.90 |
| 196 | BALF | - | - | 1.20 |
| 197 | BALF | - | - | 1.20 |
| 198 | BALF | - | - | 0.73 |
| 199 | BALF | + | + | 15.00 |
| 200 | BALF | - | - | 1.10 |
| 201 | hydrothorax | - | + | 2.17 |
| 202 | hydrothorax | - | - | 1.23 |
| 203 | hydrothorax | - | - | 1.17 |
| 204 | hydrothorax | + | + | 16.63 |
| 205 | puncture fluid | - | - | 1.03 |
| 206 | sputum | - | + | 1.50 |
| 207 | sputum | + | + | 12.63 |
| 208 | sputum | - | + | 1.77 |
| 209 | sputum | + | + | 11.63 |
| 210 | sputum | + | + | 13.30 |
| 211 | sputum | - | - | 1.30 |
| 212 | sputum | - | - | 0.33 |
| 213 | BALF | - | - | 0.57 |
| 214 | tissues | - | - | 0.70 |
| 215 | sputum | - | - | 0.73 |
| 216 | BALF | - | - | 0.83 |
| 217 | BALF | - | - | 0.72 |
| 218 | BALF | - | - | 0.85 |
| 219 | hydrothorax | - | - | 0.55 |
| 220 | BALF | - | - | 0.36 |
| 221 | cerebrospinal fluid | - | - | 0.99 |
| 222 | hydrothorax | - | - | 1.45 |
| 223 | BALF | - | - | 1.25 |
| 224 | sputum | - | - | 1.36 |
| 225 | sputum | - | - | 1.33 |
| 226 | BALF | - | - | 1.36 |
| 227 | sputum | - | - | 1.36 |
| 228 | BALF | - | - | 1.33 |
| 229 | BALF | - | - | 1.13 |
| 230 | BALF | - | - | 1.41 |
| 231 | BALF | - | - | 1.24 |
| 232 | BALF | - | - | 1.05 |
| 233 | hydrothorax | - | - | 0.60 |
| 234 | tissues | - | - | 0.72 |
| 235 | hydrothorax | - | - | 1.16 |
| 236 | sputum | - | - | 1.04 |
| 237 | BALF | - | - | 1.08 |
| 238 | tissues | - | - | 0.64 |
| 239 | sputum | - | - | 1.16 |
| 240 | BALF | - | - | 1.13 |
| 241 | BALF | + | + | 3.08 |
| 242 | BALF | - | - | 1.00 |
| 243 | BALF | - | - | 1.24 |
| 244 | BALF | - | - | 1.00 |
| 245 | hydrothorax | - | - | 0.89 |
| 246 | hydrothorax | - | - | 1.18 |
| 247 | sputum | - | - | 1.11 |
| 248 | sputum | - | - | 1.15 |
| 249 | sputum | - | + | 1.77 |
| 250 | sputum | + | + | 2.13 |
| 251 | sputum | - | - | 1.36 |
| 252 | BALF | + | + | 2.01 |
| 253 | sputum | - | - | 1.36 |
| 254 | sputum | - | - | 1.24 |
| 255 | sputum | - | - | 1.30 |
| 256 | urine | - | - | 1.36 |
| 257 | BALF | - | - | 1.30 |
| 258 | BALF | - | - | 1.06 |
| 259 | BALF | + | + | 2.37 |
| 260 | BALF | - | - | 1.21 |
| 261 | pus | - | + | 3.08 |
| 262 | hydrothorax | - | + | 1.95 |
| 263 | hydrothorax | - | - | 1.42 |
| 264 | sputum | + | + | 2.85 |
| 265 | sputum | - | - | 0.62 |
| 266 | BALF | - | - | 0.65 |
| 267 | BALF | - | - | 0.62 |
| 268 | BALF | + | + | 4.33 |
| 269 | BALF | + | + | 7.61 |
| 270 | BALF | + | + | 3.17 |
| 271 | BALF | + | + | 7.32 |
| 272 | BALF | + | + | 2.88 |
| 273 | BALF | - | - | 0.68 |
| 274 | BALF | + | + | 3.61 |
| 275 | BALF | - | - | 0.52 |
| 276 | BALF | - | - | 1.17 |
| 277 | BALF | - | - | 0.97 |
| 278 | BALF | - | - | 0.80 |
| 279 | BALF | - | - | 0.49 |
| 280 | hydrothorax | - | - | 0.97 |
| 281 | sputum | - | - | 0.83 |
| 282 | sputum | + | + | 4.33 |
| 283 | sputum | - | - | 0.88 |
| 284 | sputum | - | - | 1.09 |
| 285 | hydrothorax | - | - | 1.09 |
| 286 | sputum | - | - | 1.22 |
| 287 | sputum | - | - | 1.36 |
| 288 | sputum | + | + | 1.48 |
| 289 | tissues | - | - | 0.77 |
| 290 | sputum | + | - | 0.93 |
| 291 | sputum | + | + | 7.88 |
| 292 | BALF | + | + | 6.10 |
| 293 | tissues | - | - | 1.00 |
| 294 | urine | - | - | 1.10 |
| 295 | tissues | - | - | 0.99 |
| 296 | hydrothorax | - | - | 1.10 |
| 297 | tissues | - | - | 0.88 |
| 298 | tissues | - | - | 0.88 |
| 299 | cerebrospinal fluid | - | - | 1.09 |
| 300 | hydrothorax | + | + | 6.88 |
| 301 | BALF | - | - | 1.04 |
| 302 | BALF | + | + | 7.10 |
| 303 | BALF | + | + | 4.21 |
| 304 | sputum | - | - | 1.22 |
| 305 | sputum | + | + | 5.54 |
| 306 | hydrothorax | - | - | 1.16 |
| 307 | sputum | + | + | 6.77 |
| 308 | sputum | - | - | 0.99 |
| 309 | sputum | + | + | 5.21 |
| 310 | sputum | - | - | 0.93 |
| 311 | sputum | + | + | 1.60 |
| 312 | sputum | - | - | 0.82 |
| 313 | sputum | + | + | 2.16 |
| 314 | sputum | - | - | 0.96 |
| 315 | sputum | - | - | 1.00 |
| 316 | sputum | - | - | 1.08 |
| 317 | sputum | + | - | 1.12 |
| 318 | hydrothorax | - | - | 1.01 |
| 319 | hydrothorax | - | - | 0.68 |
| 320 | hydrothorax | - | - | 0.87 |
| 321 | BALF | + | + | 2.64 |
| 322 | BALF | - | - | 0.96 |
| 323 | BALF | - | - | 0.56 |
| 324 | BALF | - | - | 1.39 |
| 325 | BALF | - | - | 1.08 |
| 326 | BALF | - | - | 1.28 |
| 327 | BALF | - | - | 0.85 |
| 328 | sputum | - | - | 0.52 |
| 329 | sputum | - | + | 1.85 |
| 330 | sputum | - | - | 0.93 |
| 331 | sputum | - | - | 1.13 |
| 332 | sputum | + | + | 3.05 |
| 333 | hydrothorax | - | - | 0.81 |
| 334 | urine | - | - | 1.00 |
| 335 | BALF | + | - | 0.96 |
| 336 | BALF | - | - | 0.85 |
| 337 | BALF | + | + | 8.42 |
| 338 | BALF | - | - | 0.80 |
| 339 | BALF | + | + | 4.64 |
| 340 | BALF | - | - | 0.87 |
| 341 | lung puncture tissue | + | + | 4.87 |
| 342 | sputum | - | - | 0.60 |
| 343 | sputum | - | - | 0.53 |
| 344 | sputum | - | - | 0.49 |
| 345 | sputum | - | - | 0.56 |
| 346 | sputum | - | - | 0.62 |
| 347 | sputum | - | - | 0.87 |
| 348 | sputum | + | + | 4.42 |
| 349 | sputum | - | - | 0.89 |
| 350 | sputum | + | + | 4.42 |
| 351 | urine | - | - | 0.82 |
| 352 | urine | - | - | 1.02 |
| 353 | BALF | - | - | 1.09 |
| 354 | BALF | - | - | 0.69 |
| 355 | BALF | - | - | 0.76 |
| 356 | BALF | - | - | 0.87 |
| 357 | BALF | - | - | 1.11 |
| 358 | BALF | - | - | 0.47 |
| 359 | BALF | - | - | 0.53 |
| 360 | BALF | + | - | 0.56 |
| 361 | puncture fluid | - | - | 0.83 |
| 362 | hydrothorax | - | - | 1.30 |
| 363 | hydrothorax | - | + | 1.53 |
| 364 | puncture fluid | - | - | 1.03 |
| 365 | sputum | - | - | 1.07 |
| 366 | sputum | + | + | 7.97 |
| 367 | sputum | + | - | 0.50 |
| 368 | sputum | - | + | 9.63 |
| 369 | sputum | - | - | 0.50 |
| 370 | sputum | + | + | 11.30 |
| 371 | sputum | - | - | 0.53 |
| 372 | sputum | - | - | 0.80 |
| 373 | BALF | - | - | 0.90 |
| 374 | BALF | - | - | 0.60 |
| 375 | BALF | - | - | 0.93 |
| 376 | BALF | - | - | 1.30 |
| 377 | BALF | - | - | 1.33 |
| 378 | BALF | + | + | 2.03 |
| 379 | BALF | - | - | 0.63 |
| 380 | BALF | - | - | 1.30 |
| 381 | tissues | + | + | 8.63 |
| 382 | hydrothorax | + | + | 11.30 |
| 383 | sputum | - | - | 0.80 |
| 384 | sputum | - | - | 0.60 |
| 385 | BALF | - | + | 1.60 |
| 386 | BALF | - | - | 1.03 |
| 387 | BALF | + | + | 5.54 |
| 388 | BALF | - | - | 0.79 |
| 389 | sputum | - | - | 0.90 |
| 390 | BALF | - | - | 1.03 |
| 391 | sputum | + | + | 6.88 |
| 392 | BALF | - | - | 1.03 |
| 393 | hydrothorax | - | - | 0.94 |
| 394 | BALF | - | - | 0.90 |
| 395 | BALF | + | + | 3.43 |
| 396 | hydrothorax | - | + | 1.88 |
| 397 | BALF | - | - | 1.00 |
| 398 | sputum | - | - | 0.68 |
| 399 | tissues | - | - | 0.79 |
| 400 | BALF | - | - | 0.87 |
| 401 | BALF | - | - | 0.84 |
| 402 | BALF | - | - | 1.01 |
| 403 | BALF | - | - | 1.08 |
| 404 | BALF | - | - | 1.01 |
| 405 | BALF | - | - | 1.18 |
| 406 | BALF | - | - | 1.01 |
| 407 | hydrothorax | - | - | 1.43 |
| 408 | sputum | - | - | 0.68 |
| 409 | sputum | + | - | 1.40 |
| 410 | sputum | + | + | 3.63 |
| 411 | sputum | - | - | 1.21 |
| 412 | sputum | - | - | 1.10 |
| 413 | sputum | - | + | 5.06 |
| 414 | sputum | - | - | 0.77 |
| 415 | sputum | + | + | 4.25 |
| 416 | sputum | + | + | 7.06 |
| 417 | sputum | - | - | 0.94 |
| 418 | sputum | + | + | 2.85 |
| 419 | sputum | - | - | 1.02 |
| 420 | sputum | - | - | 1.02 |
| 421 | sputum | - | - | 0.77 |
| 422 | sputum | - | + | 1.94 |
| 423 | hydrothorax | - | - | 0.52 |
| 424 | hydrothorax | - | - | 0.38 |
| 425 | sputum | + | - | 0.83 |
| 426 | ascites | - | - | 0.63 |
| 427 | lung puncture tissue | - | - | 0.77 |
| 428 | tissues | - | - | 0.73 |
| 429 | BALF | - | - | 1.06 |
| 430 | BALF | - | - | 0.98 |
| 431 | BALF | - | - | 0.81 |
| 432 | BALF | + | + | 4.52 |
| 433 | BALF | - | - | 0.77 |
| 434 | BALF | - | + | 2.18 |
| 435 | sputum | + | + | 3.90 |
| 436 | sputum | - | - | 0.78 |
| 437 | sputum | - | - | 0.73 |
| 438 | sputum | - | - | 1.22 |
| 439 | sputum | + | + | 5.23 |
| 440 | urine | - | + | 1.85 |
| 441 | hydrothorax | - | - | 0.82 |
| 442 | hydrothorax | - | - | 0.87 |
| 443 | BALF | - | - | 0.68 |
| 444 | BALF | + | - | 1.32 |
| 445 | sputum | - | - | 1.18 |
| 446 | BALF | - | - | 1.12 |
| 447 | BALF | - | - | 1.17 |
| 448 | sputum | + | - | 1.20 |
| 449 | sputum | - | - | 1.10 |
| 450 | sputum | - | - | 0.85 |
| 451 | sputum | - | - | 1.32 |
| 452 | sputum | - | - | 0.77 |
| 453 | sputum | - | - | 1.18 |
| 454 | sputum | - | - | 0.78 |
| 455 | sputum | + | + | 3.55 |
| 456 | sputum | + | + | 5.07 |
| 457 | sputum | - | - | 0.62 |
| 458 | sputum | - | - | 0.80 |
| 459 | sputum | - | - | 1.15 |
| 460 | sputum | - | - | 1.15 |
| 461 | sputum | - | - | 0.92 |
| 462 | sputum | - | - | 0.85 |
| 463 | sputum | - | + | 1.70 |
| 464 | hydrothorax | - | + | 1.55 |
| 465 | tissues | - | - | 0.65 |
| 466 | tissues | - | - | 1.15 |
| 467 | cerebrospinal fluid | - | - | 1.32 |
| 468 | BALF | - | - | 0.90 |
| 469 | BALF | - | - | 0.60 |
| 470 | BALF | - | - | 0.65 |
| 471 | BALF | - | - | 1.15 |
| 472 | BALF | - | + | 1.52 |
| 473 | BALF | - | - | 1.27 |
| 474 | BALF | - | - | 0.85 |
| 475 | BALF | - | - | 0.85 |
| 476 | BALF | - | - | 0.77 |
| 477 | BALF | - | - | 0.60 |
| 478 | urine | - | - | 0.65 |
| 479 | hydrothorax | - | - | 1.02 |
| 480 | hydrothorax | - | - | 1.33 |
| 481 | urine | - | - | 1.27 |
| 482 | sputum | - | - | 0.62 |
| 483 | sputum | - | - | 1.02 |
| 484 | sputum | - | - | 0.95 |
| 485 | BALF | - | - | 1.27 |
| 486 | BALF | - | - | 0.50 |
| 487 | BALF | - | - | 0.40 |
| 488 | BALF | - | - | 0.27 |
| 489 | BALF | - | - | 0.68 |
| 490 | urine | - | - | 0.45 |
| 491 | urine | - | - | 0.35 |
| 492 | sputum | - | - | 0.40 |
| 493 | BALF | - | - | 0.70 |
| 494 | sputum | - | - | 0.62 |
| 495 | sputum | - | - | 1.02 |
| 496 | sputum | - | - | 0.35 |
| 497 | sputum | - | - | 0.52 |
| 498 | sputum | + | + | 3.65 |
| 499 | sputum | - | - | 0.70 |
| 500 | sputum | - | - | 0.87 |
| 501 | sputum | - | - | 0.82 |
| 502 | sputum | - | - | 0.80 |
| 503 | sputum | - | - | 1.15 |
| 504 | BALF | - | - | 1.28 |
